# Digital rectal exam vs. electronic “digitized” prostate exam

**DOI:** 10.1186/s12894-025-01936-y

**Published:** 2025-11-21

**Authors:** Tabea Borde, Nicole A. Varble, Alexander Kenigsberg, Lindsey A. Hazen, Laetitia Saccenti, Peter A. Pinto, Baris Turkbey, Bradford J. Wood

**Affiliations:** 1 Center for Interventional Oncology, Radiology and Imaging Sciences, Clinical Center, National Institutes of Health, 10 Center Drive, Bethesda, MD 20892 USA; 2 Philips Healthcare, Cambridge, MA 02141 USA; 3 Urologic Oncology Branch, National Cancer Institute, National Institutes of Health, Bethesda, MD 20892 USA; 4 Henri Mondor Biomedical Research Institute, Inserm U955, Team N°18, Créteil, France; 5 Molecular Imaging Branch, National Cancer Institute, National Institutes of Health, Bethesda, MD 20892 USA; 6 Center for Interventional Oncology, Radiology and Imaging Sciences, Clinical Center, National Institute of Biomedical Imaging and Bioengineering and National Cancer Institute Center for Cancer Research, National Institutes of Health, Bethesda, MD 20892 USA

**Keywords:** Prostate, Prostatic cancer, Image-Guided biopsy, Magnetic resonance imaging, Digital rectal examination, Prostate-Specific antigen

## Abstract

**Background:**

The current era of multiparametric prostate MRI (mpMRI) and ultrasound (US)/ MRI fusion has improved early detection of clinically significant prostate cancer (csPCa) while decreasing the number of unnecessary biopsies. Hence, this study aims to define the diagnostic value of pre-biopsy digital rectal examination (DRE) for screening and detection of csPCa, when there is access to mpMRI and prostate-specific antigen (PSA).

**Methods:**

This retrospective, single-center study involved 3379 consecutive patients biopsied between 2012 and 2023 using both MRI/US-targeted fusion and systematic approaches. Biopsies were indicated because of elevated PSA levels and/or presence of cancer suspicious lesions on MRI. All patients underwent DRE and mpMRI prior to biopsy. csPCa was defined as Gleason grade group ≥2. Diagnostic performance of DRE for csPCa was compared to PSA and PSA density (PSAD) using receiver operating characteristic curves.

**Results:**

Two thousand one hundred eighty-eight (65%) patients were diagnosed with PCa and 1498 (44%) patients with csPCa. DRE was positive in 410/3379 (12%) patients. 261/410 (64%) DRE positive patients had confirmed csPCa, of which 160/261 (61%) were located in immediate proximity to the rectum. mpMRI was positive for focal lesion(s) in 1494/1498 (100%) csPCa patients. For csPCa detection, DRE had a sensitivity of 0.17, specificity of 0.92, a positive predictive value of 0.63 and a negative predictive value of 0.59. Compared to DRE (AUC 0.55 [CI95% 0.54, 0.56]), PSA (AUC 0.63 [CI95% 0.61, 0.64]) and PSAD (AUC 0.73 [CI95% 0.71, 0.74]) showed better csPCa prediction performance (both *p* < 0.001).

**Conclusions:**

Pre-biopsy DRE was found to add little to no diagnostic value for the routine diagnostic workflow in a constrained setting when PSA and mpMRI are initial screening tools for patients with suspected csPCa.

**Supplementary Information:**

The online version contains supplementary material available at 10.1186/s12894-025-01936-y.

## Background

Prostate cancer (PCa) is the most common solid-organ malignancy and the second leading cause of cancer related death among biologically male individuals in the United States in 2024 [1]. Screening with digital rectal examination (DRE) and prostate-specific antigen (PSA) remain common practices, however PSA screening has received low grades by the US Preventive Services Task Force. DRE role has evolved and is specifically excluded from the proposed research approach in their final research plan for PCa screening, as of December 21, 2023 [2]. DRE has overall shown poor diagnostic performance in detecting PCa, which raises doubt on the effectiveness and utility of using DRE as a reliable screening parameter [3, 4]. In patients with normal PSA levels, there is no relevant increase in sensitivity for clinically significant PCa (csPCa, above Gleason grade group 2 [GG≥2]) when performing DRE [5, 6]. Hence, if PSA levels are being evaluated with appropriate thresholds (e.g., 4ng/mL) to prompt biopsy consideration, it remains uncertain whether adding DRE improves detection of csPCa. Multiple studies have demonstrated variability in both the consistency and performance of DRE, with altogether low inter- and intra-observer reliability [7, 8]. DRE did not correlate with biopsy findings and pathological staging which questions its contribution to diagnosis and treatment management in PCa patients [9]. In addition, DRE may present a “significant barrier” to compliance and participation in PCa screening [10].

The rise of CT scans for appendicitis, the remote telehealth visit, and the Focal ultrasound screening has led to an increased reliance upon imaging and digital examination versus the traditional physical exam [11]. The current era of multiparametric prostate MRI (mpMRI) and ultrasound (US)/MRI fusion has improved early detection of csPCa while decreasing the number of unnecessary biopsies [12]. Although not the subject of this study, the increasing indications of mpMRI for prostate examination may lead to a reassessment of the inclusion of the digital rectal exam as part of a complete physical exam or diagnostic process.

Despite the poor diagnostic performance of DRE, low interobserver consistency, limited patient compliance, and exclusion from many clinical guidelines, DRE is still routinely performed arguably due to a lack of consensus on a definitive screening approach and limited evidence of the value of mpMRI in screening or initial diagnosis. Hence, this study aims to define the diagnostic value of pre-biopsy DRE for screening and detection of csPca, when there is access to mpMRI and PSA.

## Patients and methods

Institutional review board-approved prospective clinical trial data was retrospectively analyzed (trial registration NCT00102544, registration date 2005-01-29, registry clinicaltrials.gov). Between 2012 and 2023, 3379 consecutive adult men (≥ 18 years old) were included in the study, all of whom had an abnormality on mpMRI and underwent fusion prostate biopsy. Patients initially presented at the research institution with suspected PCa from an elevated serum PSA level (> 2.5 ng/mL), and/or an abnormal DRE, and/or an abnormality identified on mpMRI [13]. All patients provided written informed consent and underwent mpMRI, which was then followed by unblinded DRE and same-day targeted MRI/US-targeted fusion biopsy and concurrent systematic 12-core biopsy. Biopsy was indicated either because of elevated PSA levels or abnormal mpMRI results with at least one suspicious lesion detected.

### Prostate imaging and histopathologic evaluation

 3 T MRI scanners (Philips Achieva [2012–2018] or Ingenia Eliton-X [2019–2023], Best, The Netherlands; MAGNETOM Skyra, Siemens Healthcare, Erlangen, Germany [2018–2019]) with surface coils was used for MRI acquisition. Although MRI sequences and technique varied over the years of the study, T2-weighted, diffusion weighted, and dynamic contrast enhanced MRI were acquired for all patients. Target lesions and corresponding extraprostatic extension were prospectively evaluated by a radiologist with 19 years expertise in mpMRI. Patients with lesions identified on mpMRI underwent both a targeted and systematic biopsy. Patients without mpMRI lesions underwent systematic biopsies only.

Primary and secondary Gleason scores were converted into the Gleason grading group system in a ranking of 1–5 groups. csPCa was defined as GG≥2 as congruent with literature [13, 14].

### Prostate assessment

DRE was performed by different urologists who estimated the prostate size in a clinically universal ranking system from “0–4+” [15] and who screened for abnormalities. A positive DRE was defined as presence of palpable, suspicious nodules, localized firmness, and/or profound asymmetry. PCa location was identified by location of positive biopsy and then classified into anterior or posterior using mpMRI. To assess the potential impact on lesion location and extraprostatic extension, mpMRI were analyzed on 70 randomly sampled patients, which were similar to the whole study population in terms of age, PSA and prostate volume (supplemental material).

The threshold for PSA was determined at ≥4 ng/ml and for PSAD at ≥0.15 ng/mL/mL.

The volume of the prostate was calculated independently using mpMRI and US exams separately. US volume measurements were estimated and calculated using an ellipsoid formula based on maximum height (H) x width (W) x length (L) × (π/6) just before prostate biopsy procedures. mpMRI volumes were measured (semi)automatically by an experienced radiologist manually contouring and adjusting the automated segmentation of the prostate gland on axial T2-weighted images during prospective clinical prostate mpMRI interpretations.

### Statistical analysis

To evaluate the ability of DRE, PSA and PSAD to detect csPCa, sensitivity, specificity, positive (PPV) and negative predictive value (NPV) were calculated including 95% confidence intervals (CI). The Shapiro-Wilk normality test was used to test for gaussian distribution of nominal variables. Descriptive statistics were presented as median and interquartile range (IQR). Receiver operating characteristic (ROC) curve analysis was performed to assess and compare independent parameters including DRE, PSA and PSA density (PSAD, quotient of PSA level divided by MRI prostate volumes) in their prediction performance for csPCa. Area under the curve (AUC) with 95% confidence intervals (CI95%) were reported. Thresholds were calculated using Youden’s J statistic [16]. To evaluate the additional value of DRE in predicting csPCa, a multivariate analysis was performed where two models were built, with and without DRE. Variables considered for model inclusion were PSAD, Age, US and mpMRI volume, DRE and mpMRI lesion positivity. Outcome was csPCa. The Mann-Whitney U test was performed to evaluate congruity between non-gaussian distributed variables. To test the concordance between dichotomous variables, the Kendall’s tau coefficient was calculated. To examine the relationship between the categorical and nominal variables, the chi-squared test of independence was performed. Model performance was evaluated using probabilities and ROC curves. Two-tailed p-values were reported for all statistical tests. All statistical tests were computed in the open-source software R (R Foundation for Statistical Computing, Vienna, Austria. URL https://www.R-project.org/).

## Results

### Patient and tumor characteristics

Patient and tumor characteristics are presented in Table 1. Median patient age was 65 years (IQR 59–70) and median serum PSA was 6.7 ng/mL (IQR 4.6–10.1, range 0.0–2910). Calculated median PSAD was 0.12 ng/mL/mL (IQR 0.08–0.19, range 0.0–24.9). In total, 38,832 systematic biopsy cores (median [IQR] 12 [12–12]/patient) and 17,236 MRI/US-targeted biopsy cores (median [IQR] 4 [3–6]/patient) were obtained.

**Table 1:** Patient and tumor characteristics

Parameter	Patient cohort *N* (%)
No. of patients	3,379
Age (median [IQR])	65 (59–70)
PSA (ng/mL, median [IQR])	6.7 (4.6–10.1)
PSAD (ng/mL/cc, median [IQR])	0.12 (0.08–0.19)
Ultrasound based volume (mL, median [IQR])	55 (40–78)
MRI based volume (mL, median [IQR])	53 (39–75)
Number of MRI fusion biopsy core per patient (median [IQR])	4 (3–6)
Number of systematic biopsy core per patient (median [IQR])	12 (12–12)
Prostate cancer	
Yes	2,188 (65%)
No	1,191 (35%)
DRE	
Positive	410 (12%)
Negative	2,969 (88%)
DRE size category	
0	604 (18%)
1+	1,196 (35%)
2+	1,318 (39%)
3+	261 (8%)
Gleason Score in prostate cancer	
MRI/US-targeted fusion biopsy	
Grade 1, Gleason < = 6	474 (22%)
Grade 2, Gleason 7 (3 + 4)	749 (34%)
Grade 3, Gleason 7 (4 + 3)	106 (5%)
Grade 4, Gleason 8 (4 + 4)	397 (18%)
Grade 5, Gleason 9–10	90 (4%)
Systematic biopsy	
Grade 1, Gleason < = 6	695 (32%)
Grade 2, Gleason 7 (3 + 4)	609 (28%)
Grade 3, Gleason 7 (4 + 3)	105 (5%)
Grade 4, Gleason 8 (4 + 4)	262 (12%)
Grade 5, Gleason 9–10	61 (3%)

*IQR* interquartile range, *PSA* prostate-specific antigen, *PSAD* prostate-specific antigen density, *MRI* magnetic resonance imaging

In 2188/3379 (65%) patients, biopsy detected prostate cancers and in 1498/3379 (44%) patients csPCa. DRE was positive in 410/3379 (12%) patients. 261/410 (64%) DRE positive patients had confirmed csPCa, of which 160/261 (61%) were located in the immediate proximity to the rectum. In contrast, mpMRI correctly identified lesions in 1494/1498 (100%) patients with biopsy-confirmed csPCa. Of the mpMRI negative, but biopsy positive patients (4/1498 [0%]), 0/4 (0%) were detected with DRE. Positive DRE showed a weak association with higher Gleason group grades (tau = 0.2, *p* < 0.001). Extraprostatic extension was reviewed in a subset analysis which is available in supplemental material.

### Diagnostic performance of DRE

For detection of csPCa (GG ≥ 2), DRE had a sensitivity of 0.17, specificity of 0.92, a PPV of 0.63 and an NPV of 0.59. Indicating that DRE had a high rate of false negative results and a low rate of false positive results. Elevated PSA (≥ 4ng/mL) had a sensitivity of 0.87, specificity of 0.25, a PPV of 0.48 and an NPV of 0.72. In contrast to DRE, elevated PSA showed a low rate of false negative results (high sensitivity), but a higher rate of false positive results (low specificity). When considering the high prevalence of PCa in this cohort, PSA showed a lower false positive rate than DRE. Elevated PSAD (≥ 0.15 ng/mL/mL) had a sensitivity of 0.55, specificity of 0.78, a PPV of 0.67 and an NPV of 0.69.

DRE correctly identified 261/1498 (17%) csPCa patients. PSA (≥ 4ng/mL) correctly identified 1304/1498 (87%) and PSAD (≥ 0.15 ng/mL/mL) 1219/1498 (81%) csPCa patients. mpMRI detected cancer suspicious lesions in 1494/1498 (100%) csPCa which resulted in 1342/1498 (90%) positive MRI/US-targeted fusion biopsies.

When DRE and PSA results were combined, diagnostic performance for detection of csPCa (sensitivity 0.9, specificity 0.22, PPV 0.48, NPV 0.74) remained similar to PSA alone. 37/1498 (2%) of csPCa could be diagnosed additionally when combining DRE and PSA compared to PSA alone (1341/1498 [90%]), irrespective of mpMRI. The combination of positive PSA and MRI identified all patients with csPCa (1498/1498 [100%]), and therefore, the addition of positive DRE did not find any additional patients. However, for detection of patients with clinically insignificant PCa (GG = 1), the triple combination identified 1 additional patient compared to MRI and PSA alone (690 versus 689, GG = 1 PCa).

**Fig. 1:**
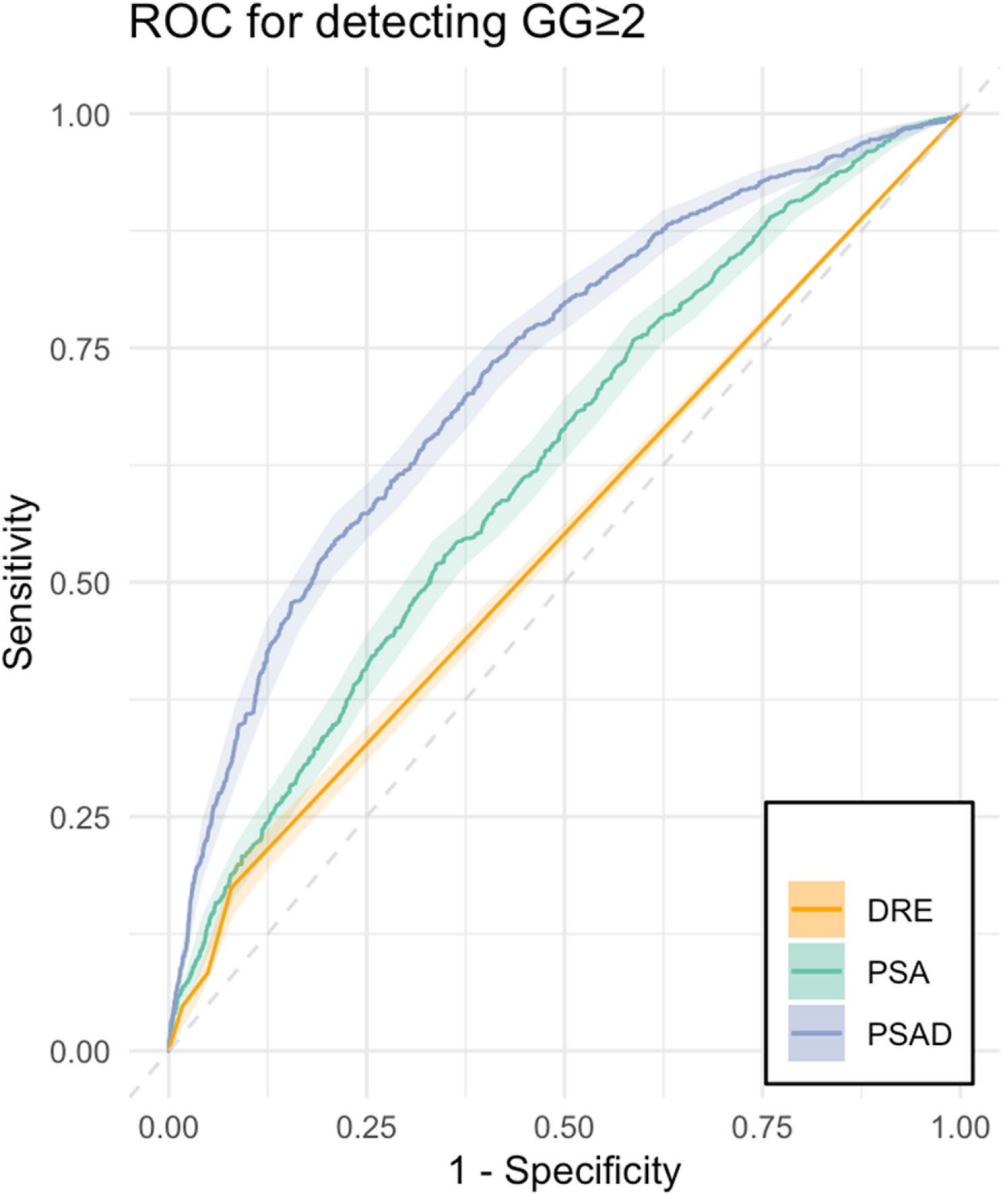
Prediction performance of DRE, PSA, and PSAD for clinically significant prostate cancer detection. Receiver operating characteristic (ROC) curves for prediction performance of clinically significant prostate cancer Gleason grade group (GG) ≥ 2 using digital rectal examination (DRE, AUC 0.55 [CI95% 0.54, 0.56]), prostate-specific antigen (PSA, AUC 0.63 [CI95% 0.60, 0.64]) and MRI-based prostate-specific antigen density (PSAD, AUC 0.73 [CI95% 0.71, 0.74]). PSA and PSAD showed better prediction performance over DRE (CI95% [−0.1, −0.05], *p* < 0.001, CI95% [−0.19, −0.15], *p* < 0.001, respectively)

Compared to DRE (AUC 0.55 [CI95% 0.54, 0.56]), PSA (AUC 0.63 [CI95% 0.60, 0.64], *p* < 0.001), and PSAD (AUC 0.73 [CI95% 0.71, 0.74], *p* < 0.001) showed better cancer prediction performance for csPCa in ROC analysis (Fig. 1).

### DRE and lesion size estimation

DRE size estimation moderately correlated with MRI and US volume measurements (both *p* < 0.001, Kendall tau = 0.41). DRE size 0 corresponded to a median MRI volume of 36 mL (IQR 27–50), size 1 + a median MRI volume of 46 mL (IQR 37–59), size 2 + 64 mL (IQR 50–80), and size 3 + 105 mL (IQR 80–132). DRE size 2 + showed the highest probability for cancer (AUC 0.66, compared to DRE size 0, *p* = 0.001). Normal size and large size (DRE size 3+) had lower prediction performance for detecting csPCa (AUC 0.57 [CI95% 0.52, 0.61], 0.60 [CI95% 0.53, 0.68] respectively). Patients with large prostates had 18% less prostate cancer prevalence compared to small sized prostates (range 73–55%).

### Multivariate analysis for determination of CsPCa

**Fig. 2:**
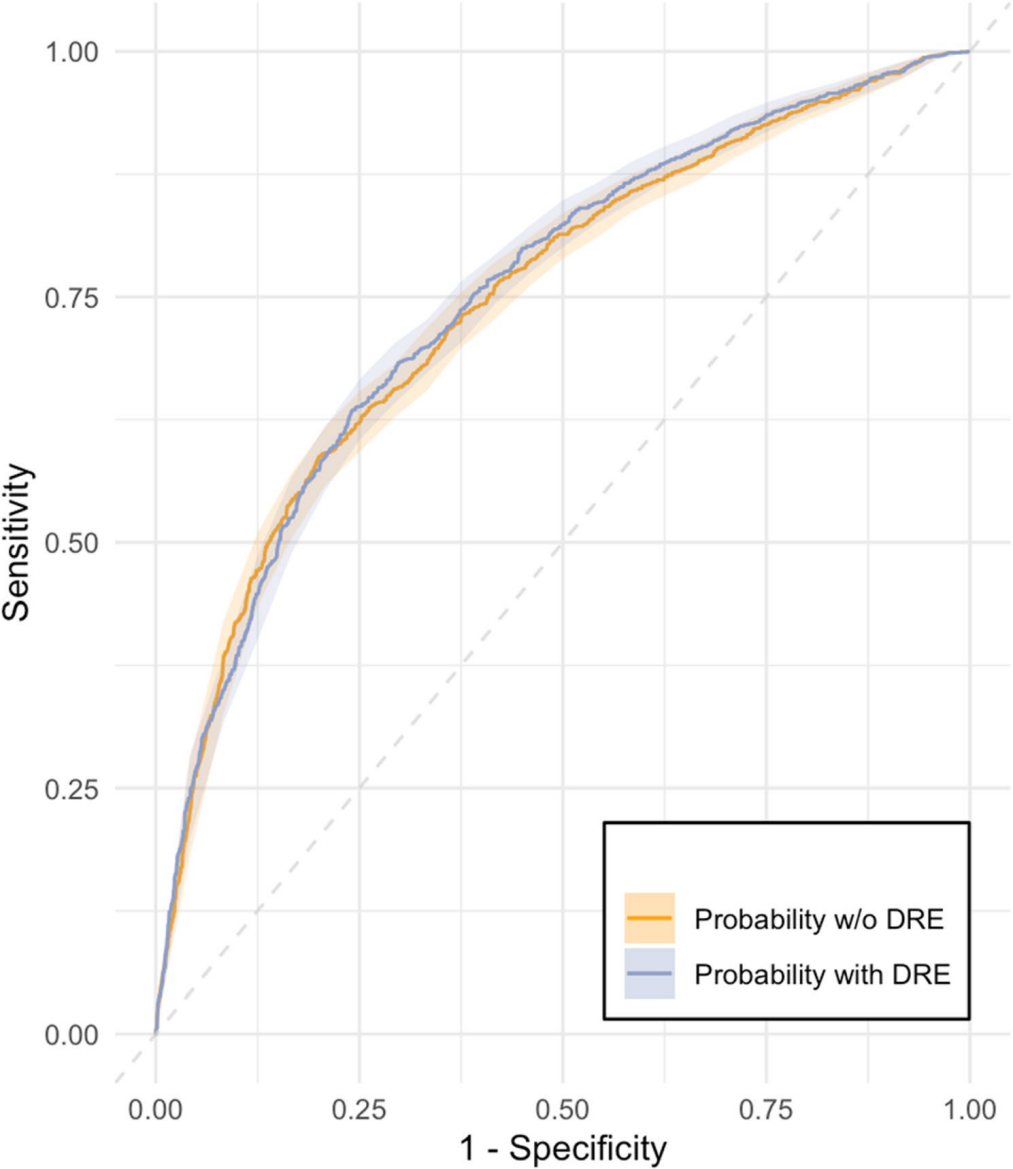
Comparison of multivariate models with/without DRE for prediction performance for clinically significant prostate cancer detection. Receiver operating characteristic (ROC) curves for prediction performance of prostate cancer Gleason grade group (GG) ≥ 2. There was no statistical difference between the probability of multivariate model including digital rectal examination (DRE, AUC 0.75 [CI95% 0.73, 0.77]) and probability of multivariate model without DRE (AUC 0.74 [CI95% 0.73, 0.76], *p* = 0.07)

To identify differences in prediction performance for detecting patient with csPCa with and without DRE, multivariate analysis was performed (Table 2). Variables included in Model#1 were PSAD, age, DRE and mpMRI lesion positivity. Variables included in Model #2 were PSAD, age, and MRI lesion positivity. In Model #2 (without DRE), Hazard Ratios did not change substantially (i.e. Hazard Ratio PSAD: Model #1 with DRE = 222 (109.5, 464.6) vs. Model #2 without DRE = 230 (113.8, 478.7). Furthermore, as analyzed by ROC curve analysis (Fig. 2), there was no statistical difference between the predictive performance of Model#1 including DRE (AUC 0.75 [CI95% 0.73, 0.77]) and Model#2 without DRE (AUC 0.74 [CI95% 0.73, 0.76], *p* = 0.07).

**Table 2:** Multivariate analysis with and without DRE

Variables	Model#1 with DRE		Model#2 without DRE	
	Odds Ratio	*P*-value	Odds Ratio	*P*-value
Age	1.05 (1.04, 1.07)	< 0.001	1.06 (1.05, 1.07)	< 0.001
PSAD	222 (109.5, 464.6)	< 0.001	229.7 (113.8, 478.7)	< 0.001
Positive MRI lesions	8.04 (2.90, 31.98)	< 0.001	8.30 (3.00, 33.01)	< 0.001
Positive DRE	2.03 (1.61, 2.56)	< 0.001		

*PSAD* prostate-specific antigen density, *MRI* magnetic resonance imaging, *DRE* digital rectal examination

### Independent predictors of CsPCa compared to DRE

**Fig. 3:**
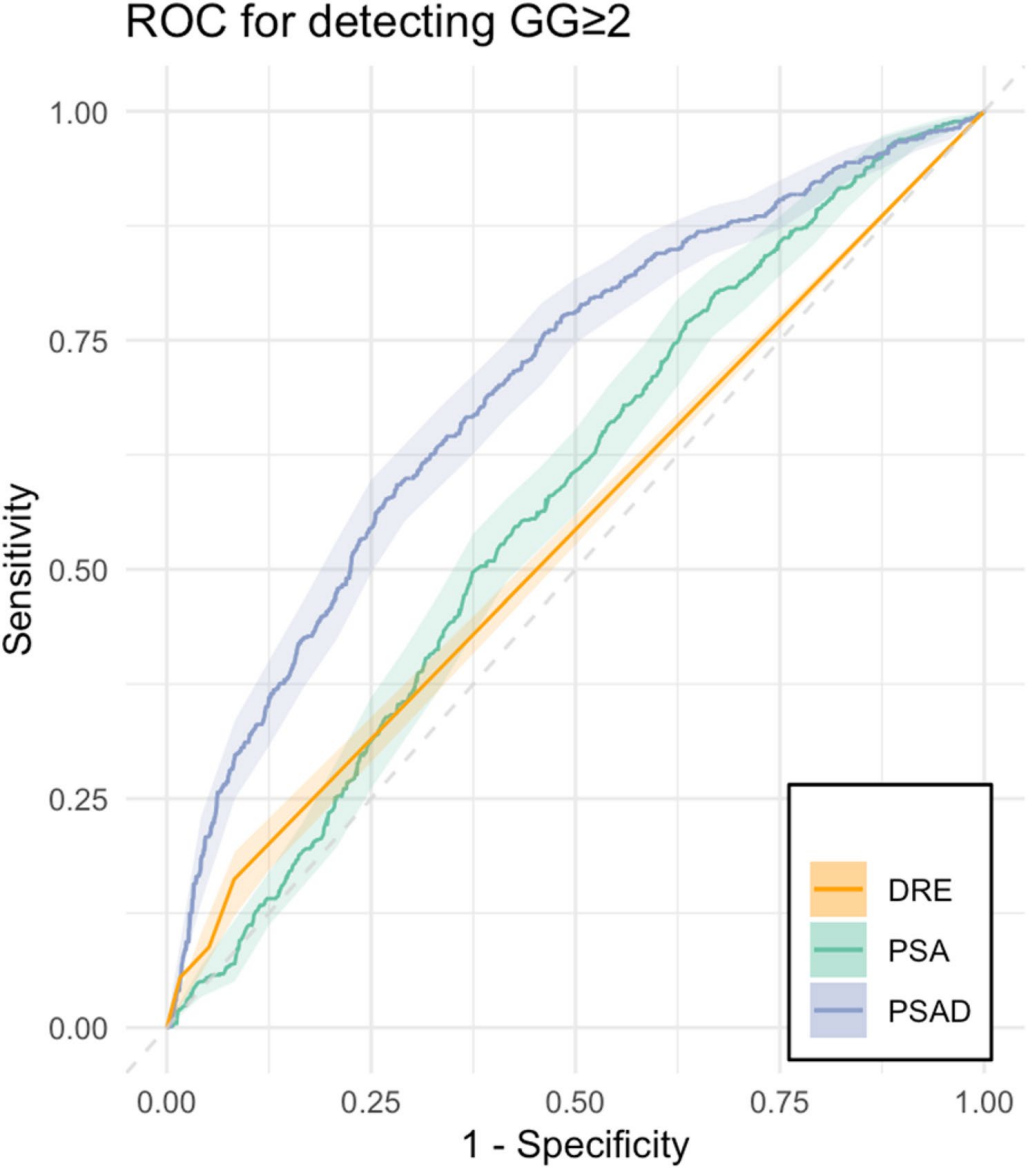
Prediction performance of DRE, PSA and PSAD for clinically significant PCa in patients with PSA < 7.5ng/mL. Receiver operating characteristic (ROC) curves for prediction performance of prostate cancer Gleason grade group (GG) ≥ 2 using digital rectal examination (DRE, AUC 0.54 [CI95% 0.52, 0.56]), PSA (AUC 0.57 [CI95% 0.55, 0.60]) and MRI-based prostate-specific antigen density (PSAD, AUC 0.70 [CI95% 0.68, 0.73]). PSA and PSAD showed better prediction performance over DRE (CI95% [−0.1, −0.05], *p* = 0.03, CI95% [−0.17, −0.11], *p* < 0.001)

When analyzing independent predictors of csPCa and to compare their diagnostic performance to DRE in this specific cohort, Youden’s J statistical method identified 7.5 mg/mL (AUC 0.63, corresponding sensitivity 0.51, specificity 0.66) as the threshold that best differentiated patients with and without csPCa. In a subset analysis including patients with PSA levels < 7.5ng/mL, DRE showed a similar prediction performance for csPCa to the total cohort (AUC 0.54 [CI95% 0.52, 0.56]). Including patients with < 7.5ng/mL PSA values, the prediction performance of PSA (AUC 0.57 [CI95% 0.55, 0.60]) was still better than that of DRE (*p* = 0.03). The better prediction performance of PSAD remained similar (AUC 0.70 [CI95% 0.68, 0.73], *p* < 0.001, Fig. 3). In patients with PSA levels < 7.5ng/mL, and < 4ng/mL, the detection rate of DRE remained similar with 16% (116/717), and 19% (36/188), respectively, compared to 17% in the total cohort. There was no difference in PSA levels between patients with positive and negative DRE (*p* = 0.10).

In patients with normal PSAD (< 0.15 ng/mL/mL), DRE showed a similar low prediction performance as the total cohort (AUC 0.54 [CI95% 0.53, 0.56]), with a detection rate of 16% (111/674).

## Discussion

The role of DRE is diminishing in the era of mpMRI and fusion biopsy for the diagnostic workup of prostate cancer, as evidenced by its omission from research agendas in the ongoing USPSTF updated screening grades. In a highly selected set of patients where mpMRI was integrated into the routine clinical workup, DRE had little to no additional value as a diagnostic tool. The exact role of DRE in other more broad populations remains ill-defined. However, DRE may remain a critical diagnostic tool in patients with advanced disease (including extraprostatic extension) without access to regular screening nor mpMRI.

In this study in a tightly constrained scenario of conditions, unblinded DRE size estimations moderately correlated with MRI and ultrasound volume measurements. Larger prostates were found to have a higher risk for csPCa than smaller prostates [17]. However, performance as a solitary screening tool was well below multiple other approaches. The prediction performance of DRE for csPCa showed less efficacy than PSA or PSAD alone and did not identify additional patients when combined with PSA and mpMRI. Altogether, DRE had low sensitivity, but high specificity and high PPV for csPCa diagnosis in this highly selected cohort. Several studies reported similar low sensitivity and high specificity values for DRE, however with slightly lower PPVs [18–20]. This might be due to differing patient selection biases, and patient inclusion criteria. The definition and interpretation of DRE is variable as well, including subjective criteria such as asymmetry, irregularity and induration [20]. Since DRE is subject to high inter- and intra-observer variability [4, 7, 21], it may be difficult to determine and standardize objective criteria for DRE across physicians and practices.

The significance of positive DRE results remains patient and cohort-specific, which may challenge reliable interpretation. In this high prevalence study cohort, 64% patients with positive DREs had csPCa. When considering the palpability of tumors, 15% of those patients had tumors in the anterior gland, not in immediate proximity to the rectum. Only limited data is available correlating mpMRI findings versus location of DRE findings [22]. DRE in the post-treatment setting was not studied here. The combination of DRE, PSA and mpMRI did not identify additional csPCa patients than PSA and mpMRI alone, in this tightly constrained population. These findings suggest that DRE adds little additional diagnostic value in the presence of mpMRI. Also, the extensive informational gain of mpMRI over DRE is undeniable since DRE merely provides tactile, perhaps more superficial information. Although not studied here, DRE may remain helpful in the assessment of tumor operability, particularly for locally advanced disease (clinical stage T3/T4) [23]. In this study, however, there was no correlation between DRE results and higher Gleason group grades. Recent studies comparing performance of mpMRI and DRE for PCa staging found that mpMRI improved detection of stage ≥ T3a as well as of early PCa compared to DRE [24, 25]. Furthermore, mpMRI showed better performance metrics [26] and performed better than DRE even when conducted by the most experienced clinicians [27]. If DRE does not contribute to the diagnostic workup of csPCa, it also has little influence on the treatment management of csPCa.

Although speculative, DRE might prove cost effective if it increased the sensitivity of a PSA test, particularly in detecting aggressive tumors with low PSA levels that might otherwise be missed. When analyzing independent predictors of csPCa in this specific cohort, DRE did not show an advantage in low-level PSA cancers. Several studies have looked at the utility of DRE as a screening test for csPCa in low PSA levels, none of which found a favorable detection performance for DRE in patients with PSA levels below 4ng/mL [5, 19, 22, 27]. In this study, the diagnostic prediction performance for csPCa did not show a difference with or without including DRE similar to a recent meta-analysis including 85,738 patients [20]. Due to its low detection rate and high potential to lead to unnecessary biopsy and overdiagnosis, DRE is not recommended in the primary care setting and is not recommended as a screening parameter in men < 45 years [4, 28]. Compliance with prostate screening and diagnosis carries specific sensitivities and hurdles to implementation of any guideline. When surveyed, 27% of men felt ashamed and 16% of men feared a DRE beforehand [29]. During DRE, 73% of men experienced pain and discomfort [30]. In addition, only 78% of men would regularly participate in a screening that included both DRE and PSA [10]. PSA-only screening would thus detect more cancers with fewer negative biopsies due to higher use, irrespective of a theoretical positive screen rate or higher sensitivity from combined screening methods. Therefore, it is unknown what element of compliance might be related to reticence or similar factors in patients. These findings emphasize a potential emotional barrier to seek medical care and engage in prostate screening programs including DRE.

This study has some limitations. It is critical not to overstate or assume generalization. Specifically, the results are retrospective and from a highly pre-selected cohort, and thus cannot be extrapolated to broader populations, nor to the general use of DRE for screening in any specific demographic population or set of conditions, specifically in any community setting. Most importantly, this clinical trial focused on patients with mpMRI-visible lesions that could be targeted during fusion biopsy or elevated PSA levels. These strict criteria in a single center study define major patient selection bias with high cancer detection rates in all cohorts. Additionally, all critical steps in the study including DRE, MRI interpretation, volume measurements, prostate biopsies, and pathology evaluations were conducted or supervised by experts in the field, in a clinical center dedicated entirely to translational research, which may limit generalizability or reproducibility of findings in the community or any other setting. DREs were generally performed just prior to biopsy procedures without blinding to the MRI findings, which may create bias across groups. However, this bias might also be expected to enhance the sensitivity of DRE, so may not diminish the study findings. An additional confounding factor is that when patients were referred for further workup after primary point of care screening elsewhere, that initial DRE (existence and result) was not recorded or known.

## Conclusions

In conclusion, pre-biopsy DRE was found to add little to no diagnostic value for the routine diagnostic workflow in a constrained setting when PSA and multiparametric prostate MRI are initial screening tools for patients with suspected csPCa. Although DRE may carry less value with the advent of MRI and fusion biopsy, prospective study of this physical exam staple is required before rushing to judgement on its clinical utility for any given scenario.

## Supplementary Information

Supplementary Material 1

## Data Availability

The data sets generated during and/or analyzed during the current study are available from the corresponding author on reasonable request.

## Abbreviations

DRE: Digital rectal examination
PCa: Prostate cancer
csPCa: Clinically significant prostate cancer
PSA: Prostate–specific antigen
PSAD: PSA density
mpMRI: Multiparametric prostate MRI
GG≥2: Gleason grade group above 2
PPV and NPV: Positive and negative predictive value
CI: Confidence intervals
IQR: Interquartile range
ROC: Receiver operating characteristic curve
AUC: Area under the curve
