# Supplementary material for: Digital rectal exam vs. electronic “digitized” prostate exam
Source: BMC Urol. 2025 Nov 21;26:2. doi: 10.1186/s12894-025-01936-y (PMC12771905; doi:10.1186/s12894-025-01936-y)
Supplement: Supplementary file 1 — Supplementary Material 1 [file 12894_2025_1936_MOESM1_ESM.docx]

# **Supplemental material**

Extraprostatic extension (EPE) is a strong independent predictor for recurrence after radical prostatectomy, and therefore, may indicate the potential need for treatment intensification [1, 2]. Seventy patients with positive DRE were randomly chosen for a subset analysis in which lesion location and extraprostatic extension were analyzed on mpMRI. This subset had similar descriptive features and demographics to the total cohort of patients with positive DRE, with regards to nearly identical age (median 65 years [IQR 60–70]), PSA levels (median 6.7 ng/ml [4.6–10.2]) and prostate volume (median 54 ml [IQR 39–77]). In this subset, 80/90 (89%) patients had csPCa. 46/90 (51%) patients had EPE adjacent to the rectum seen on mpMRI review. Median lesion size on mpMRI was 1.6 cm (IQR 1.1–2.2). All lesions were categorized as PI-RADS 5 and median Gleason grade group was 4 (8 [4+4]).

This subset analysis demonstrates that only half of the patients with positive DRE results have EPE adjacent to the rectum which would be expected to be palpable on DRE. This finding might add to the questionability of the reliability of DRE.

1. Ball MW, Partin AW, Epstein JI. Extent of Extraprostatic Extension Independently Influences Biochemical Recurrence-free Survival: Evidence for Further pT3 Subclassification. Urology. 2015;85(1):161-4. DOI: https://doi.org/10.1016/j.urology.2014.08.025.

2. Jeong BC, Chalfin HJ, Lee SB, et al. The Relationship Between the Extent of Extraprostatic Extension and Survival Following Radical Prostatectomy. European Urology. 2015;67(2):342-6. DOI: https://doi.org/10.1016/j.eururo.2014.06.015.
